# A Multi-Institutional, Retrospective, Observational Study on Administration Status and Safety of In-Hospital Oral Selenium Preparation in Pediatric Patients Predominantly Suffering from Gastrointestinal Disease

**DOI:** 10.3390/nu16183142

**Published:** 2024-09-17

**Authors:** Jumpei Saito, Eiji Suzuki, Keiko Kobayashi, Keisuke Doi, Yosuke Miwa, Setsuko Ihara, Kei Nakai, Miki Akabane

**Affiliations:** 1 Department of Pharmacy, National Center for Child Health and Development, 2-10-1, Okura, Setagayaku 157-8535, Tokyo, Japan; 2 Department of Pharmacy, Nagano Children’s Hospital, 3100, Toyoshina, Azumino 399-8288, Nagano, Japan; 3 Department of Pharmacy, University Hospital, Kyoto Prefectural University of Medicine, 465 Kaji-cho, Kawaramachi-dori Hirokoji-agaru, Kamigyoku, Kyoto 602-8566, Kyoto, Japan; 4 Department of Pharmacy, Aichi Medical Treatment and Education Center Central Hospital, 713-8 Kamiyacho, Kasugai 480-0392, Aichi, Japan; 5 Shizuoka Children’s Hospital, 860 Urushiyama, Aoiku, Shizuoka 420-8660, Shizuoka, Japan; 6 Miyagi Children’s Hospital, 4-3-17 Ochiai, Aobaku, Sendai 989-3126, Miyagi, Japan

**Keywords:** selenium, gastrointestinal diseases, pediatric safety

## Abstract

Objectives: Selenium deficiency in patients with gastrointestinal diseases treated with long-term central venous nutrition is a clinical problem. Only injectable selenium is approved in Japan, and oral selenium preparations are prepared in hospitals from reagents, but their efficacy and safety are unknown. Methods: We conducted a retrospective study investigating the relationship between selenium administration and oral selenium formulations and adverse events. Results: In this study, 239 selenium-treated cases and 220 selenium-untreated cases adjusted for patient background were selected as a reference group. The median (interquartile range, IQR) age was 1.3 (0.4–4.4) and 1.3 (0.3–4.5) years, respectively; gastrointestinal diseases were most common in 110 (46.0%) and 104 (47.3%) cases. The median (IQR) duration of treatment or observation with oral selenium was 446 (128–1157) and 414 (141–1064) days, respectively. The median (IQR) dose per body weight at the maintenance dose was 2.6 (1.7–3.9) μg/kg, and the median (IQR) serum selenium concentration at the maintenance dose was 8.5 (7.0–10.6) μg/mL within the upper tolerated dose limit and approximately the reference range. There was no difference in selenium dose, serum selenium concentration, or serum-selenium-concentration-to-dose ratio (C/D ratio) for adverse events. The incidence of adverse events was compared with that of patients not treated with selenium. Conclusions: An oral selenium preparation administered below the upper tolerated dose limit can be used effectively and safely in pediatric patients.

## 1. Introduction

Selenium is an essential trace element for maintaining human physiological functions [1]. With the increase in long-term central venous nutrition therapy cases, selenium deficiency cases have also been reported in Japan. In a study, the selenium contained in food is organic selenium, and selenocysteine (Sec) and selenomethionine (SeM) are known [2,3,4]. Foods with a high selenium content include seafood, meat, eggs, bread, pasta, and Chinese noodles made from North American wheat. Although few studies have rigorously identified the molecular species of selenium in animal foods, the presence of selenoproteins with Sec is known in animals, including fish. Children with diseases who have difficulty eating a regular diet, i.e., patients on total parenteral nutrition, will become selenium-deficient if selenium supplementation is not administered. Selenium deficiency has also been reported in patients using enteral nutrition products, specialized milk, and therapeutic milk that does not contain selenium. The underlying diseases of patients receiving enteral or intravenous nutrition include severe physical and mental disabilities, inflammatory bowel disease, short bowel disease, and congenital disorders. The main clinical symptoms of selenium deficiency are the whitening and deformation of nails, muscle pain, arrhythmia, and cardiomyopathy, which causes heart failure [2,3,4]. In addition, neurological disorders, skin diseases, and alopecia have also been reported [5,6,7,8]. Cardiomyopathy presents as dilated cardiomyopathy, which may progress to heart failure in severe cases [9,10]. The onset of selenium deficiency and toxicity is due to the process from absorption to excretion after selenium intake. Selenium taken in through food and water is absorbed efficiently in the gastrointestinal tract via the transport system, and, ultimately, more than 90% is absorbed in the gastrointestinal tract and transferred to the blood. It is finally converted to HSe- ions, released into the plasma and taken up by various tissues, where it is used in the biosynthesis of selenoproteins. The biosynthesized selenoproteins are involved in antioxidant effects and thyroid hormone metabolism and play an essential role in maintaining normal physiological functions. In addition, the HSe-ions that are produced are quickly excreted. In patients using parenteral nutrition, enteral nutrition, and unique milk formulas, selenium intake is considered insufficient. In patients with renal failure, in addition to dietary restrictions, selenium absorption is thought to be reduced. Consumption is believed to be increased, resulting in a decrease in blood selenium concentration [4,5,6,7,8,9,10].

In Japan, selenium injection (Aselend^®^ Injection 100 μg/2 mL) was approved and marketed in 2019 [11]. However, oral selenium preparations are not approved as a pharmaceutical product at this stage. Therefore, they are made as an in-hospital preparation from reagents at each medical institution [12]. This preparation at our center is about 5000 bottles of 7 mL/bottle (used up in one week), used for about 100 inpatient and outpatient pediatric patients per year (surveyed from April 2022 to March 2023). In a survey of 39 pediatric facilities affiliated with the Japan Association of Children’s Hospitals and Related Institutions (JACHRI), we received responses from 31 facilities. We found that 15 facilities manufacture an in-house formulation of oral selenium solution from reagents (results not yet published). Many patients using oral selenium preparations are restricted from using dietary supplements containing selenium because of their disease.

Recently, an oral selenium product has been attempted to be developed by Fujimoto Pharmaceutical Co., Ltd. (Matsubara, Japan), and a Phase III clinical trial has been completed (clinical trial number jRCT2031220210, enrolled in October 2022, completed June 2024) [13]. The pediatric patients to be included in this clinical trial are six months or older, and the number of pediatric patients to be further evaluated (unknown at this stage) is expected to be small. For the injectable selenium product Aselend^®^ Injection, only 26 pediatric patients between the ages of 1 and 11 years were enrolled out of 63 patients in the Phase III clinical trial. Furthermore, the observation period is up to 13 months. The safety of the injectable selenium formulation in children under one year of age and the long-term safety in all patients continues to be a post-marketing surveillance [11]. Although oral selenium products have been used in medical facilities for a long time, there have been only a few reports on the efficacy and safety of oral selenium products in pediatric patients in Japan (2–15 cases) [14,15,16] and efficacy and safety evaluations have not yet been conducted on a large number of patients.

Adverse events reported in clinical trials of injectable selenium products and previous reports of oral selenium supplementation include nasal hemorrhage, dermatitis, keratitis, renal dysfunction, sinus tachycardia, increased serum creatine phosphokinase, and hyperbilirubinemia [4,17]. In addition, renal dysfunction, headache, seizures, cardiomyopathy, vomiting, diarrhea, dermatitis, alopecia, and neuritis have been reported as adverse events due to environmental exposure (soil and tap water), chemical exposure (gun bluing agent, and selenium toxicity in mismanufactured selenium dietary supplements) [18,19,20,21,22,23,24,25,26,27,28,29,30,31,32,33].

This study aimed to investigate the status of the preparation and administration of oral selenium preparations prepared from reagents and to examine the safety of selenium and the relationship between selenium dosage and serum concentrations in pediatric patients administered oral selenium preparations.

## 2. Materials and Methods

### 2.1. Study Design

The design of this study was a multi-center, retrospective observational study that collected information from six children’s hospitals. The National Center for Child Health and Development and five hospitals that prepare oral selenium preparations as an in-hospital formulation were set as study sites to collect information on pediatric patients who received oral selenium preparations.

### 2.2. Subjects of Analysis

Two cohorts were studied to investigate retrospectively the status of oral selenium product administration and the association between adverse events and selenium administration; in both cohorts, patients who were taking selenium-containing products (enteral nutrition, supplements, etc.) other than oral selenium products as in-hospital formulations were excluded from the analysis.

Selenium intake by food was not included in the analysis of the association between selenium administration and serum selenium concentration because the exact amount of selenium intake could not be assessed.

#### 2.2.1. Cohort 1: Investigation of Selenium Prescription Status and Serum Selenium Concentration

In Cohort 1, patients’ age, disease, duration of treatment, selenium dosage, and measured serum selenium concentration were evaluated as selenium prescription status. Inclusion criteria for Cohort 1 were as follows: (1) patients were between 0 and 18 years of age at the time of oral selenium prescription, (2) patients received an in-hospital oral selenium preparation, (3) at least one reference serum selenium concentration test result was available, and (4) age and weight at the time of selenium administration and serum selenium concentration measurement were known. Information on the oral selenium preparation method and information on eligible patients at each medical facility were collected retrospectively from the electronic medical records. In addition, cases of apparent selenium intake other than selenium intravenous and in-hospital preparations such as elemental diet were excluded.

#### 2.2.2. Cohort 2: Analysis of the Association between Selenium Administration and the Occurrence of Adverse Events

Cohort 2 investigated whether selenium administration increases the occurrence rate of adverse events. For comparison of the occurrence rate ratio due to selenium administration, as a nested cohort, the cohort before selenium administration in the eligible patients who were administered selenium was compared with the “selenium-untreated cases” regarding adverse event occurrence status. The inclusion criteria for Cohort 2 were as follows: (1) patients had to be between 0 and 18 years of age at the time of oral selenium administration, (2) patients had to be treated with an in-hospital oral selenium preparation, (3) the baseline laboratory tests before selenium administration were available, (4) the “selenium-treated” cases had to have at least one reference serum selenium concentration test result, and (5) patient age and weight at the time of selenium administration or at the time of adverse event assessment were known. In addition, cases of apparent selenium intake other than selenium intravenous and in-hospital preparations such as elemental diet were excluded. The occurrence rate of adverse events per 1000 person-years was calculated to correct for the selenium administration and observation duration. For group comparisons, the number of “selenium-treated” and “selenium-untreated” cases and the distribution of patient background factors, such as age, proportion of gender, and disease category, were selected before cohort analysis of adverse events to ensure that the distribution of these factors was comparable.

### 2.3. Survey Period

Patients who received selenium from January 2008 to 31 March 2023 at six children’s hospitals were selected, and information was collected on the selected patients back to the start of selenium administration. In Cohort 1 and Cohort 2, “selenium-treated cases”, patients were chosen from 1 April 2016 to 31 March 2023, when serum selenium concentration measurement became covered by insurance.

### 2.4. Survey Items

#### 2.4.1. Selenium Dosage and Serum Selenium Concentration: Cohort 1

The selenium concentration was evaluated using the age-specific reference values for serum selenium levels defined in the Guidelines for the Treatment of Selenium Deficiency 2018 (Japanese Society of Clinical Nutrition) [4]. The selenium dose per body weight and the number and percentage of stated cases in the groups with serum selenium concentration lower than the reference value range (Low), within the reference value range (Normal), and higher (High) were calculated.

#### 2.4.2. Investigation of the Occurrence of Adverse Events Due to Administration of Oral Selenium Products: Cohort 2

Age, body weight, selenium dose, disease, serum selenium concentration, duration of administration, and status of administration of selenium products other than in-hospital selenium preparations (intravenous selenium products and selenium from nutritional supplements) were collected. Safety information was collected using the International Statistical Classification of Diseases 10 (ICD 10) code and clinical laboratory values. Adverse events were collected from adverse reactions observed in clinical trials and from literature information on renal impairment, gastrointestinal disorders, diarrhea, vomiting, alopecia, neuritis/neuropathy, keratitis, dermatitis, seizures, nose bleeds, myocardial infarction, headache, sinus tachycardia, high creatine kinase (CK), and hyperbilirubinemia. For each adverse event, items recorded after administration of oral selenium products were collected; for high CK and hyperbilirubinemia, laboratory values were referenced. The published “Pediatric Clinical Laboratory Reference Values (National Center for Child Health and Development)” was used as the reference values for clinical laboratory values [34] (https://www.sogo-igaku.co.jp/lec_in_ped/0302.html, accessed on 31 August 2024). Laboratory values were graded using the Common Terminology Criteria for Adverse Events (CTCAE version 5.0, Japan Clinical Oncology Group version) as a baseline before selenium administration [35]. The laboratory values were considered abnormal when there was an increase of grade 2 (moderate) or higher.

### 2.5. Statistical Analysis

Analyses were conducted to determine if patient background (age), selenium dosage, and selenium serum concentration contribute to developing adverse events and whether oral selenium products increase the incidence of adverse events. To explore factors associated with adverse event occurrence, we compared selenium doses, serum selenium concentrations, and patient background in the occurrence group (cases) and the non-occurrence group (controls). In this comparative analysis, no adjustment for patient background was made in the crude analysis.

To assess whether oral selenium treatment increased the occurrence of adverse events, the number of cases of adverse events in the “selenium-treated cases” and the “selenium-untreated cases” selected by adjusting for patient background was divided by the total time (years) post-treatment until the event occurred and then multiplied by 1000 to calculate the incidence rate per 1000 person-years.

In the comparison between the two groups, after confirming the normal distribution (Shapiro–Wilk test), the Mann–Whitney U test was performed if the distribution did not follow the normal distribution, and Welch’s *t*-test was performed if the distribution did follow the normal distribution, but the variances were different. For cases with equal variances, a Student t-test was performed. The significance level was set at 5% for all tests. All statistical analyses were performed using IBM SPSS statistics 30.0.0 (IBM Japan, Ltd., Tokyo, Japan).

### 2.6. Ethical Consideration

All researchers in this study conducted their research under the “Declaration of Helsinki” and the “Ethical Guidelines for Life Sciences and Medical Research Involving Human Subjects”. The National Center for Child Health and Development Ethics Review Committee reviewed the study in writing from the viewpoint of ethical, scientific, and medical appropriateness before and during the study period (Approval No. 2023-020, approved on 22 May 2023).

In this research, only information collected during routine medical examinations will be extracted from electronic medical records and used. Therefore, individual consent was not obtained from the study subjects or their custodians. Instead of obtaining individual consent, a summary of the study was posted in visible locations within each research institution and on the official website, and research subjects and their guardians who wished to refuse to participate in the study were informed that they could withdraw from the study by informing us of their intention.

## 3. Results

### 3.1. Preparation Method of Oral Selenium Preparation

Five of the six surveyed hospitals prepared selenium solutions (5–50 mL per container) with a final concentration of 10–50 μg/mL (as selenium) by adding sterile distilled water to selenite and sterilizing it with high-pressure steam. After preparation, the solution was stored in a light-shielded, cool place, and the required amount was weighed, or a pre-prepared container was dispensed to the patient. The remaining hospital diluted sodium selenite with lactose to prepare a selenium powder with a final concentration of 300 μg/g (0.03%). To confirm the dilution process, the product was colored with 0.1% brilliant blue.

### 3.2. Patient Inclusion by Each Cohort

**Figure 1:**
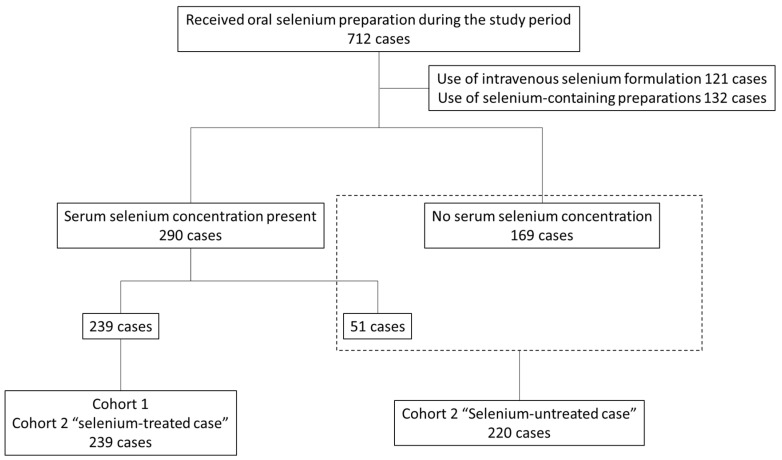
Data inclusion for each cohort.

The case inclusion process for each cohort is shown in Figure 1. A total of 712 cases were selected at the medical institutions where the study was conducted during the study period. Of these, 121 patients who received intravenous selenium products and 132 who received selenium-containing products other than oral selenium products (enteral nutrition, supplements, etc.) were excluded, leaving 472 patients for analysis; 390 of the 472 patients had serum selenium concentration data, and 69 had no data. The “selenium-treated cases” in Cohorts 1 and 2 included 239 cases from the 390 cases with serum selenium concentrations. A total of 220 patients were selected for the “selenium-untreated cases” in Cohort 2, including 69 patients with no selenium serum concentration data and 151 patients from the 390 patients with selenium serum concentration data (of the cases with selenium serum concentration data, 29 were included in Cohort 2, “selenium-untreated cases”, because their body weights were unclear at the time of administration).

### 3.3. Patient Background

The patient background of the 239 patients included as selenium-treated cases in Cohort 1 and 2 and the 220 patients included as selenium-untreated cases in Cohort 2 is shown in Table 1. The proportion of males was 56.3% and 55.9%, respectively, and the median (interquartile range, IQR) age was 1.3 (0.4–4.4) and 1.3 (0.3–4.5) years, respectively. Hospitalizations accounted for 70.7% and 71.4% of patients in each group. The median (IQR) duration of oral selenium therapy or observation was 446 (128–1157) days in the selenium-treated cases and 414 (141–1064) days in the selenium-untreated cases. The number of prescriptions for oral selenium products in selenium-treated cases was 6081. The median (IQR) duration of the maintenance dose was 151 (60.3–494.5) days when the same dose per body weight was continued as the “maintenance dose. The median (IQR) dose per body weight at the maintenance dose was 2.6 (1.7–3.9) μg/kg, and the median (IQR) serum selenium concentration at the maintenance dose was 8.5 (7.0–10.6) μg/dL. Gastrointestinal diseases were the most common primary diseases in each group, accounting for 110 (46.0%) and 104 (47.3%) of the selenium-treated and selenium-untreated cases, respectively. Congenital diseases were the next most common, accounting for 85 (35.6%) and 79 (35.9%) cases, respectively. The most common congenital diseases were congenital heart disease, organ failure, malformations, and metabolic diseases, most of which were due to genetic factors. Most patients treated with in-hospital selenium preparations received parenteral or enteral component nutrition therapy without selenium and had difficulty taking a regular diet.

### 3.4. Cohort 1: Analysis of Serum Selenium Concentration and Factors Causing Adverse Events

#### 3.4.1. Serum Selenium Concentration

Serum selenium concentrations measured in Cohort 1 were evaluated regarding age-specific reference values (Table 2). The number of cases that were classified as “Low”, “Normal”, or “High” was 172 (83.9%), 182 (88.8%), and 41 (20.0%), respectively, and the most significant number of cases were within the reference values. However, one in five cases had selenium concentrations above the upper reference limit. The median (IQR) age at the time of the initial evaluation was 1.2 (0.5–4.0), 2.0 (0.9–5.4), and 4.6 (2.7–7.1) years for “Low”, “Normal”, and “High”, respectively, with the younger age group being lower than the reference value and the older age group being higher than the reference value. The median (IQR) duration of treatment at the time of the initial evaluation was 35 (30–79) days, 84 (30–224) days, and 356 (141–792) days for each group, respectively, and many cases were below the reference value when the duration of treatment was short. In addition, the duration of administration was longer in cases where the upper reference limit was exceeded. By inpatient and outpatient status, 29.7%, 29.1%, and 39.0% of the cases were classified as “Low”, “Normal”, and “High”, respectively, and the number of cases classified as “High” was higher in the outpatient setting.

#### 3.4.2. Comparison of Serum Selenium Concentration with Adverse Events

Based on ICD 10 codes and laboratory values, we investigated the occurrence of adverse events and compared the measured serum selenium concentrations (Table 3). In this case-control analysis, controls adjusted for patient background factors were not included for adverse event cases, and a crude analysis was performed by comparing adverse event cases and controls within Cohort 1. The occurrence of renal failure, gastrointestinal disorders, diarrhea, vomiting, alopecia, neuritis/neuropathy, keratitis, dermatitis, seizures, epistaxis, myocardial infarction, headache, sinus tachycardia, high CK level, and cases with hyperbilirubinemia were investigated. The occurrence of each adverse event was 54 cases of renal failure, 64 cases of gastrointestinal disorders, 34 cases of diarrhea, 36 cases of vomiting, 5 cases of alopecia, 5 cases of neuritis/neuropathy, 6 cases of keratitis, 13 cases of dermatitis, 8 cases of convulsions, 5 cases of nasal hemorrhage, 2 cases of myocardial infarction, 15 cases of headache, 2 cases of sinus tachycardia, 78 cases of abnormal (high) CK, and 49 cases of hyperbilirubinemia. Compared to the control group, there were no statistically significant differences in selenium dosage, serum selenium concentration, or the ratio of serum selenium concentration to dosage (C/D ratio) for any adverse events.

### 3.5. Cohort 2: Comparison of the Occurrence of Adverse Events Due to Oral Selenium Administration

The occurrence rate of adverse events and 95% confidence intervals were calculated for the “selenium-untreated cases”, which were referred to as the nested cohort before oral selenium administration, and the “selenium-treated cases” which were administered oral selenium products (Table 4). The occurrence of adverse events was investigated in the same manner as in Cohort 1. Using the “selenium-untreated cases” as reference, the occurrence rate ratios (95% confidence interval) exceeded 2 for alopecia, keratitis, seizures, myocardial infarction, and high CK, and the respective occurrence rate ratios (95% confidence interval) were 3.1 (0.2–41.4) for alopecia, 2.4 (0.1–60.2) for keratitis, 3.8 (0.4–39.1) for seizures, myocardial infarction 3.3 (0.4–29.1), and high serum CK 5.5 (0.5–63.9). However, the occurrence of any of these adverse events did not increase in the selenium-treated cases compared with the selenium-untreated cases.

The occurrence of each adverse event was also investigated by disease (Supplementary Materials Table S1). The cumulative occurrence of adverse events per case was higher in patients with infectious diseases (4.7 in the exposure case and 6.1 in the reference case) and in patients with pediatric cancer (4.0 in the exposure case and 4.1 in the reference case). However, there was no relationship between adverse events and underlying diseases in cases where selenium was administered and where selenium was not administered. The number of cases for each disease in this survey was limited, and further surveys limited to specific diseases may be necessary.

## 4. Discussion

The status of serum selenium concentrations was investigated in Cohort 1 regarding the duration of treatment, dosage, and patient backgrounds. The median (IQR) serum selenium concentration was 8.5 (7.0–10.6) μg/dL, and the median (IQR) maintenance dose was 2.6 (1.7–3.9) μg/kg once daily. In two Japanese Phase III clinical trials of selenium for injection, the initial dose was set at 2 μg/kg, followed by 1–5 μg/kg depending on serum concentrations [17]. The resulting serum selenium concentrations were reported to be 7.66 ± 1.95 μg/dL and 6.57 ± 2.37 μg/mL, respectively. Therefore, we believe that selenium administered orally as an oral solution prepared from a reagent as an in-hospital preparation can be administered at the same dosage level as an injectable formulation and still achieve comparable serum concentrations.

In addition, when referring to the dose, duration of administration, and patient backgrounds, a high percentage of serum selenium concentrations evaluated within 30 days of administration were below the reference range. In clinical trials, a low rate of cases reached within the reference range immediately after the start of dosing and reached the reference range after repeated daily dosing. In clinical trials, the percentage of patients achieving within the reference range at four weeks after the continuation of injectable selenium was roughly 60.0–63.8% [17]. In contrast, the rate in this study was 83.9% at 30 days. The clinical study included patients with hyposelenemia at the start of the study, and it is possible that the pre-dose serum selenium concentration was simply higher in this study and that the median dose in this study was 2.7 μg/kg, which was higher than the clinical study dose.

The median (IQR) ages of the Low, Normal, and High groups were 1.2 (0.5–4.0), 2.0 (0.9–5.4), and 4.6 (2.7–7.1) years, respectively, indicating that some factors might have contributed to the lower serum selenium concentration in the younger age group. The median (IQR) dose was 2.7 (1.7–3.7) μg/kg, 2.9 (1.8–4) μg/kg, and 3.3 (2.6–4.6) μg/kg, respectively, and the high serum selenium concentration group showed a tendency to have a higher IQR, suggesting that the dose also affected the serum selenium concentration. Considering that the median (IQR) number of days of administration was 65 (160–320) days and the median dose (IQR) was 1.9 (2.8–4.1) μg/kg/day in cases with the less-than-2-years-old group (115 cases, 596 concentrations), it is possible that factors other than dose and number of days of administration caused the decrease in serum selenium concentration. It was likely that differences in selenium absorption and excretion processes (selenium is not easily absorbed or excreted) or disease may have had an effect. However, no studies on the disposition of selenium in children by age were conducted, and no conclusions could be drawn.

Although the number of patients was small, one in five patients had serum selenium concentrations exceeding the upper reference limit at least once during treatment [4,36]. The number of patients who exceeded the upper reference limit was higher in older patients (median age 4.6 years) and in long-term patients (median duration of treatment 356 days). The proportion of outpatient management cases was also high, with 29.7% and 29.1% in the Low and Normal groups, respectively, while the High group was slightly higher, at 39.0%. Factors contributing to the high serum selenium concentrations observed in the outpatient group included the inability to adjust serum selenium concentrations due to the less frequent testing for serum selenium confirmation and the intake of selenium other than the in-hospital formulation during outpatient management (i.e., food or other undeclared selenium-containing substances). In addition, oral selenium preparations were administered with the monitoring of serum selenium concentrations, resulting in 88.8% of cases ending up within the reference range. Unfortunately, the date of the first serum selenium concentration measurement varied from case to case, and only a few patients were measured within 30 days, making it impossible to correctly assess how long it would take for the serum selenium concentration to reach the reference range.

Next, in Cohort 1, age, serum selenium concentration, dose, C/D ratio, and duration of treatment were compared between the case group that experienced an adverse event and the control group that did not. Although the analysis in this study did not adjust for disease or patient background between cases and controls, there were no differences in age, selenium dose, serum selenium concentration, C/D ratio, or duration of treatment between the cases and controls.

In Cohort 2, a comparison of adverse events with cases not treated with selenium who were matched for age, disease, and other patient backgrounds revealed no adverse events with an occurrence ratio significantly greater than 1.

In the case–control comparison of Cohort 1 and the comparison of selenium-treated and selenium-untreated cases in Cohort 2, a certain percentage of the adverse events of interest occurred in the selenium-treated cases. However, there was no increase in the rate of occurrence in the selenium-treated cases compared with the selenium-untreated cases. Several explanations might account for this absence of difference: First, most of the patients in this study had selenium doses below the upper tolerable limit and serum selenium concentrations below the upper reference limit [4]. The highest selenium doses in this study were 200 µg/day in 3- and 10-year-olds, with the highest serum selenium concentrations of 12.8 µg/dL and 10.9 µg/dL, respectively. The highest serum selenium concentration was 25.8 μg/dL in a 10-year-old child receiving 75 μg/day. The maximum tolerated dose recommended by medical guidelines and dietary reference intakes is based on the results of epidemiological studies of past health hazards. The minimum health hazard-free dose of selenium in children, adults, and the elderly is 800 µg/day when hair and nail fragility and loss is used as an indicator of chronic selenium poisoning and is 13.3 µg/kg with an uncertainty factor of 2 applied to 6.7 µg/kg body weight/day (100 µg/day for 1–5 years, 150 µg/day for 6–7 years, 200 µg/day for 8–9 years, 250 µg/day for 10–11 years, 350 µg/day for males and 300 µg/day for females for 12–14 years, and 400 µg/day for males and 350 μg/day for females) [4,37]. There is no recommendation for infants (children under one year of age) because of insufficient information, and only a recommended intake of 15 µg/day has been provided. Adverse events reported as selenosis did not occur at the usual recommended dose or upper tolerable limit [38] but were reported when 900 µg/day was taken, exceeding the maximum safe intake of 400 µg [39]. Serum selenium concentrations reported in adverse event cases to date have far exceeded the serum selenium concentration and tolerance limits set at 320 µg/dL (approximately 5000 µg ingested) [23] and 324.8 µg/dL (2144 µg ingested). In an epidemiological study of high selenium dose exposure due to incorrect supplement manufacturing, diarrhea, alopecia, nail discoloration, and nausea were reported at an estimated selenium intake of 41,749 μg/day [24]. In this previous study, serum selenium concentrations were evaluated in eight patients and were reported to be 75.1 μg/dL. Some observational studies with plasma selenium concentrations of up to 91.3 μg/dL have also shown no adverse events [40]. It has also been suggested that excessive selenium intake, even below the current upper tolerance limit, may promote type 2 diabetes and total mortality. However, the accuracy of this suggestion has been questioned [30,41,42]. Referring to the safety information in the drug package inserts for injectable selenium products, it is clearly stated that no adverse events have been observed at standard doses of selenium intake in the United States, Canada, Australia, Europe, and other countries. These facts suggested that one of the reasons the incidence of adverse events did not increase in the present study was that there were no cases in which the upper tolerable limit of selenium dosage or the upper reference limit of serum selenium concentration was exceeded.

The second reason is the possibility of reflecting the characteristics of the effects of selenium on human health. The relationship between deficiency and excess selenium as a trace element is U-shaped, and it has been indicated that both a deficiency in and excess selenium intake have adverse effects on health [43,44]. The cases in this study were all from populations that required oral selenium medication (not necessarily patients diagnosed with selenium deficiency by measuring serum selenium concentrations). The possibility that the patients were in a selenium-deficient state cannot be ruled out since the “selenium-untreated cases” to which the occurrence of adverse events was compared and the time before selenium administration in the population that required selenium administration were investigated. Before selenium administration, serum selenium concentrations were not evaluated, and “selenium deficiency” could not be excluded from the “selenium-untreated cases”.

In the present study, selenium exposure was below the upper tolerable limit previously indicated, and most of the selenium was safely administered when focusing on serum selenium concentrations. On the other hand, it is also true that there are still issues to be addressed regarding the upper tolerance limits for selenium [45]. As is generally the case when setting upper tolerance limits or reference intake levels for any dietary constituent, the following observations are worth considering for experimental designs to provide a robust risk assessment model: first, that selenium metabolism is affected by gender and genetic variation [46], and, second, that selenium-induced adverse events are subclinical and can occur even when selenium is supplemented below the upper tolerated dose limit [41]. Third, the evaluation of exposure-induced adverse event development should be differentiated between organic selenium (selenium yeast, selenopolysaccharides, selenoproteins, etc.), which is used in foods and supplements, and selenite, which is used in oral and injectable selenium formulations. It has been reported that both organic and inorganic selenium cause adverse events with high-dose ingestion [47] but with different toxicity doses and profiles [48,49,50].

This study investigated the administration and safety of an oral selenium preparation made from selenite as an in-hospital formulation in children. The dosage of oral selenium to maintain serum selenium concentrations within the reference range was similar to that of the injectable selenium formulation. The occurrence of adverse events was not increased in the study population. Selenium doses were below the upper tolerated dose limit, and serum selenium concentrations were within the reference range compared to patients not treated with selenium. The limitations of this study are that the frequency of adverse events in selenium-treated patients was not adjusted for patient background in the case and control groups, and patients with selenium deficiency were not excluded based on serum selenium concentrations in the selenium-untreated patients. Moreover, although selenium intake other than that of in-hospital preparations was collected, including the use of intravenous selenium preparations, selenium intake through enteral and elemental nutrition, and information on selenium-containing food intake based on declarations, the details of selenium intake through tap water and food could not be investigated. Selenium requirements and consumption by the body vary with developmental stage, and it may be necessary to examine the development of adverse events by age.

Oral selenium products will be developed as drugs in the future, but clinical trials are expected to collect efficacy and safety information on only some pediatric patients, significantly younger patients. We hope the information in this survey will help supplement the safety information in the pediatric population.

## 5. Conclusions

Selenium preparations prepared as an in-hospital formulation were administered to a pediatric patient population, primarily patients with gastrointestinal diseases, and all patients received doses below the upper tolerable limit and used them safely without an increased occurrence rate of adverse events. The future development of oral selenium formulations for pediatric use is warranted.

## Figures and Tables

**Table 1:** Patients’ background.

	Selenium-Treated Case	Selenium-Untreated Case
Number of patients (male, %)	239 (135, 56.5%)	220 (123, 55.9%)
Median age (IQR) ^a^	1.3 (0.4–4.4)	1.3 (0.3–4.5)
Inpatient/outpatient classification (%)	Inpatient 169 (70.7%) Outpatient 70 (29.3%)	Inpatient 157 (71.4%) Outpatient 63 (28.6%)
Median days of administration or observation (IQR) ^b^	446 (128–1157)	414 (141–1064)
Number of prescriptions	6081	-
Median days to maintenance dose (IQR) ^c^	151 (60.3–494.5)	-
Median dose per body weight at maintenance dose (μg/kg) ^c^	2.6 (1.7–3.9)	-
Median selenium concentration at maintenance dose (μg/dL) ^c^	8.5 (7–10.6)	-
Disease classification		
Gastrointestinal diseases (%)	110 (46.0%)	104 (47.3%)
Crohn’s disease of the small and large intestine	26	24
Food protein-induced gastroenteritis	18	17
Ulcerative colitis	18	20
Hirschsprung’s disease	13	11
Eosinophilic gastroenteritis	9	8
Primary immunodeficiency disease	9	10
Other enterocolitis	7	4
Short bowel syndrome	7	8
Intestinal obstruction	3	2
Congenital diseases (%)	85 (35.6%)	79 (35.9%)
Congenital heart disease	23	22
Liver failure, biliary stasis, biliary atresia	12	9
Renal failure, nephrotic syndrome, cystic kidney	10	7
Urinary tract and renal malformations	6	7
Congenital metabolic anomalies	5	8
Bone, blood vessel, lymphangioma	3	4
Congenital muscle disease	2	2
Biliary stasis/biliary atresia	1	2
Other congenital malformations	15	11
Other congenital anomalies (excluding malformations)	8	7
Cerebral neurological disease (%)	15 (6.8%)	11 (5.0%)
Acute encephalopathy	6	5
Epilepsy	3	2
Hydrocephalus	3	2
Cerebral palsy	3	2
Infection (%)	10 (4.2%)	8 (3.6%)
Sepsis/infection/abscess	6	4
Pneumonia/Bronchitis	4	4
Childhood cancer (%)	7 (2.9%)	7 (2.9%)
Hematological malignancy	3	4
Solid malignancy	4	3
Hypersensitivity diseases (%)	5 (2.1%)	6 (2.7%)
Food allergies	3	4
Atopic dermatitis	2	2
Other diseases (%)	7 (2.9%)	5 (2.3%)
Developmental delay	2	1
Endocrine dysfunction	2	1
Guillain–Barré syndrome	1	1
Diaphragmatic hernia	1	1
Airway stenosis	1	1
Eating disorders	1	1

^a^ Age was indicated at the first visit to the hospital for the selenium-untreated group and on the first day of treatment for the selenium-treated group. ^b^ Duration of oral selenium formulation in the selenium-treated group and duration of observation in the selenium-untreated group were indicated. ^c^ “At maintenance dose” was defined as the most frequent treatment period with the same selenium dose.

**Table 2:** Age, selenium dose, and duration of treatment by serum selenium concentration.

	Low	Normal	High
Case number ^a^	172 (83.9%)	182 (88.8%)	41 (20.0%)
Number of outpatients	51 (29.7%)	53 (29.1%)	16 (39.0%)
Number of observed serum selenium concentrations (%)	619 (34.8%)	1066 (60.0%)	93 (5.2%)
Median age (years, IQR) ^b^	1.2 (0.5–4)	2 (0.9–5.4)	4.6 (2.7–7.1)
Median dose (µg/kg/day, IQR)	2.7 (1.7–3.7)	2.9 (1.8–4)	3.3 (2.6–4.6)
Median treatment days at the time of evaluation (IQR)	35 (30–79)	83.5 (30–223.5)	356 (141–792)
Median serum selenium concentration (µg/mL)	5 (3.9–6)	8.6 (7.8–10.6)	15.8 (15.2–16.9)

Cases below the lower limit of the age-appropriate selenium standard were classified as “Low”, those within the standard as “Normal”, and those exceeding the upper limit as “High”. ^a^ Including duplicates. ^b^ Age at the time of initial evaluation.

**Table 3:** Age, dose, serum selenium concentration, and duration of selenium administration in selenium-treated cases with and without adverse events.

Adverse Effects	ICD10	Case Number	Median Age (IQR) ^a^	Dose (µg/kg/day)	Serum Selenium Concentration (µg/dL)	C/D Ratio	Dosing Periods
Renal failure	N289/N12/N259/N10/N142	54	4.1 (1.8–7.7)	2.6 (1.8–3.4)	9.3 (7.4–12.4)	3.6 (1.9–6.6)	378.5 (193.8–1166.8)
		185	2.1 (0.8–5.8)	2.6 (1.7–4)	8.9 (6–10.9)	3.4 (1.9–5.7)	125 (30.3–354.5)
Gastrointestinal disorders	A09/K297/K291/K529	64	3 (1.7–6.1)	2.5 (1.9–3.9)	8.9 (6.5–11.5)	3 (1.9–5.4)	406.5 (103–848.8)
		175	2.2 (0.8–5.9)	2.6 (1.6–3.8)	9 (6.7–11)	3.6 (1.9–6)	128 (34.8–338)
Diarrhea	K529/K591	34	3.6 (1.3–6.1)	3.1 (2–4.6)	9.4 (5–12)	3 (1.6–5)	227 (74.5–876.3)
		205	2.3 (0.8–6.2)	2.6 (1.6–3.9)	8.7 (6.4–11)	3.5 (1.9–5.9)	138.5 (34–426.5)
Vomiting	R11	36	2.9 (1.7–5.8)	2.4 (1.9–4.2)	9 (5.6–10.7)	2.6 (1.8–5.8)	366 (123–709.8)
		203	2.4 (0.8–6.2)	2.6 (1.6–3.8)	9.1 (6.7–11.3)	3.5 (2–6.1)	146 (40.8–430.8)
Alopecia	L639	5	7.4 (3.2–11.2)	2.9 (1.5–3)	8.1 (7.5–8.6)	4.1 (2.8–5)	204 (58–279)
		234	2.5 (1–6.2)	2.6 (1.7–4)	9 (6.7–11.3)	3.4 (1.9–5.9)	174 (46.5–481.5)
Neuritis/Neuropathy	G629/G64	5	2 (2–4)	5 (1–6)	8 (4–19)	2.5 (2.5–4.1)	2.5 (2.5–4.1)
		234	2.6 (1–6.3)	2.6 (1.7–4)	9 (6.7–11.3)	3.5 (2–6)	168 (49–492)
Keratitis	H169	6	6.4 (3.5–7.5)	2.9 (2–3.1)	12.8 (7.6–13.8)	5.3 (4.4–6.8)	412 (360.3–1061.5)
		233	2.5 (0.9–6.2)	2.6 (1.7–4)	9 (6.7–11.3)	3.4 (1.9–5.9)	157.5 (45.8–455.8)
Dermatitis	L309/R21	13	2.1 (0.9–3.2)	2.9 (2.5–3.2)	9.8 (7.7–11.7)	3.1 (2.4–5.3)	217 (170–680)
		226	2.9 (1–6.5)	2.6 (1.6–3.9)	9 (6.7–11.3)	3.5 (1.9–6)	174 (46.5–496.5)
Convulsions	R568/R252	8	4.2 (2.2–6.6)	3.7 (3.3–4.8)	8.5 (8.1–9.4)	2.4 (1.6–3.1)	245.5 (149.8–468)
		231	2.4 (0.9–6.2)	2.6 (1.6–3.9)	9 (6.7–11.3)	3.5 (1.9–6.1)	157.5 (44.3–459.8)
Nasal hemorrhage	R040	5	9 (5.9–10.3)	3.2 (3.1–5.6)	8.6 (5.8–9.1)	2.6 (1.8–2.9)	1059 (370–2210)
		234	2.5 (1–6.2)	2.6 (1.7–3.9)	9 (6.7–11.3)	3.5 (2–6)	168 (49–481.5)
Myocardial infarction	I219	2	0.6 (0.5–0.6)	5.3 (4.2–6.3)	9 (7.4–10.5)	1.7 (1.7–1.8)	123.5 (106.3–140.8)
		237	2.8 (1–6.3)	2.6 (1.7–3.9)	9 (6.7–11.3)	3.5 (1.9–6)	175 (50.3–493)
Headache	R51	15	8.7 (3.4–13.2)	2.5 (1.7–3.7)	8.6 (7.8–10.4)	4.2 (1.8–5.9)	526 (181–1371.5)
		224	2.3 (0.9–5.9)	2.6 (1.7–3.9)	9 (6.7–11.3)	3.5 (1.9–6.1)	156 (45–446)
Sinus tachycardia	R000	2	2.3 (2–2.6)	1.7 (1.6–1.8)	8.9 (6.9–10.9)	5 (4.1–5.8)	140 (83–197)
		237	2.7 (1–6.3)	2.6 (1.7–4)	9 (6.7–11.3)	3.4 (1.9–5.9)	171 (48.5–493)
Abnormal CK ^b^	R748	78	5.7 (4.3–8.9)	1.8 (2.6–4.2)	5.1 (7.8–11.1)	1.6 (2.7–5.9)	30.5 (117–263)
		133	9 (6–12.4)	1.8 (2.7–3.7)	7.3 (9–11.1)	2.1 (3.2–5)	132.3 (456–1170.8)
Hyperbilirubinemia ^c^	R798	49	4 (1.6–7.5)	2.9 (2.1–4.6)	9.8 (7.1–12)	3.3 (1.6–5.7)	321.5 (131.5–989.5)
		160	3.1 (1.5–7.4)	2.6 (1.8–3.7)	9 (7.3–10.8)	3.3 (2–5.2)	411 (130–947)

^a^ Age at the onset of the adverse event or the end of the observation period. ^b^ 28 cases had missing CK values. ^c^ 30 cases had missing serum bilirubin levels.

**Table 4:** The ratio of occurrence of adverse events in selenium-treated to selenium-untreated cases.

Adverse Effects		Event Case	Total Observed Person Years	Incidence Rate (Cases/1000 Person-Years)	Ratio (95% Confidence Interval)
Renal failure	Reference	51	15,024	3.39	-
	Exposure	54	38,771	1.39	0.48 (0.04–6.02)
Gastrointestinal disorders	Reference	41	25,035	1.64	-
	Exposure	64	35,945	1.78	1.17 (0.08–16.64)
Diarrhea	Reference	40	16,427	2.44	-
	Exposure	34	15,221	2.23	1.26 (0.11–14.63)
Vomiting	Reference	29	10,873	2.67	-
	Exposure	36	17,821	2.02	1.34 (0.12–14.84)
Alopecia	Reference	2	1369	1.46	-
	Exposure	5	1374	3.64	3.11 (0.23–41.44)
Neuritis/Neuropathy	Reference	6	1140	5.26	-
	Exposure	5	1169	4.28	0.99 (0.11–8.67)
Keratitis	Reference	4	5272	0.76	-
	Exposure	6	4062	1.48	2.35 (0.09–60.23)
Dermatitis	Reference	6	7080	0.85	-
	Exposure	6	7017	0.86	1.37 (0.04–43.31)
Convulsions	Reference	21	8976	2.34	-
	Exposure	21	3348	6.27	3.78 (0.36–39.2)
Nasal hemorrhage	Reference	1	457	2.19	-
	Exposure	5	6223	0.8	0.44 (0.02–9.73)
Myocardial infarction	Reference	5	1612	3.1	-
	Exposure	2	247	8.1	3.3 (0.37–29.19)
Headache	Reference	1	570	1.75	-
	Exposure	15	13,016	1.15	0.73 (0.04–13.45)
Sinus tachycardia	Reference	3	431	6.96	-
	Exposure	2	280	7.14	1.22 (0.16–9.5)
Abnormal CK	Reference	73	31,030	2.35	-
	Exposure	78	19,465	4.01	5.54 (0.48–63.86)
Hyperbilirubinemia	Reference	85	18,222	4.66	-
	Exposure	49	39,469	1.24	0.54 (0.03–8.97)

## Data Availability

Restrictions apply to the availability of some or all data generated or analyzed during this study to preserve patient confidentiality or because they were used under license. The corresponding author will detail the restrictions and any conditions under which access to some data may be provided upon request.

## Supplementary Materials

The following supporting information can be downloaded at: https://www.mdpi.com/article/10.3390/nu16183142/s1, Table S1: The occurrence of adverse events in selenium-treated to selenium-untreated cases by underlying diseases.
