# Supplementary material for: A Multi-Institutional, Retrospective, Observational Study on Administration Status and Safety of In-Hospital Oral Selenium Preparation in Pediatric Patients Predominantly Suffering from Gastrointestinal Disease
Source: Nutrients. 2024 Sep 17;16(18):3142. doi: 10.3390/nu16183142 (PMC11435100; doi:10.3390/nu16183142)
Supplement: Supplementary file 1 [file nutrients-16-03142-s001.zip › nutrients-3203972-supplementary.pdf]

nutrients

MDPI

Supplementary Table S1. The occurrence of adverse events in selenium-treated to selenium-untreated cases by underlying diseases.

Adverse Effects		Event case	Gastrointestinal Diseases (%)	Congenital Diseases (%)	Cerebral neurological disease (%)	Infection (%)	Childhood cancer (%)	Hypersensitivity Diseases (%)	Other Diseases (%)
Whole	Reference (event/case)	701 (3.2)	332 (3.2)	216 (2.7)	47 (4.3)	49 (6.1)	30 (4.3)	19 (3.2)	8 (1.6)
	Exposure (event/case)	699 (2.9)	340 (3.1)	213 (2.5)	46 (3.1)	47 (4.7)	28 (4)	17 (3.4)	8 (1.1)
Renal failure	Reference	51	25	16	2	3	3	2	0
	Exposure	54	26	17	3	2	3	2	1
Gastrointestinal disorders	Reference	41	23	16	0	1	1	0	0
	Exposure	64	26	31	0	2	3	1	1
Diarrhea	Reference	40	21	11	5	1	1	1	0
	Exposure	34	20	10	4	0	0	0	0
Vomiting	Reference	29	13	13	2	0	0	0	1
	Exposure	36	20	15	1	0	0	0	0
Alopecia	Reference	2	1	0	1	0	0	0	0
	Exposure	5	3	0	1	0	1	0	0
Neuritis/Neuropathy	Reference	6	2	0	2	2	0	0	0
	Exposure	5	1	0	2	0	1	1	0
Keratitis	Reference	4	1	0	1	2	0	0	0
	Exposure	6	3	1	0	1	0	1	0
Dermatitis	Reference	6	2	0	1	1	2	0	0
	Exposure	6	2	0	1	1	1	1	0
Convulsions	Reference	21	12	4	3	1	1	0	0
	Exposure	21	8	6	7	0	0	0	0
Nasal hemorrhage	Reference	1	0	0	0	0	1	0	0
	Exposure	5	2	1	1	1	0	0	0
Myocardial infarction	Reference	5	1	1	1	1	1	0	0
	Exposure	2	1	0	1	0	0	0	0
Headache	Reference	1	1	0	0	0	0	0	0
	Exposure	15	4	4	1	2	2	1	1
Sinus tachycardia	Reference	3	1	1	1	0	0	0	0
	Exposure	2	0	1	1	0	0	0	0
Abnormal CK	Reference	73	34	19	1	12	3	2	2

	Exposure	78	33	22	2	11	1	7	2
Hyperbiliru-binemia	Reference	85	46	27	2	5	5	0	0
	Exposure	49	33	13	1	1	1	0	0
